# Cellular and Molecular Players in the Tumor Microenvironment of Renal Cell Carcinoma

**DOI:** 10.3390/jcm12123888

**Published:** 2023-06-07

**Authors:** Francesco Lasorsa, Monica Rutigliano, Martina Milella, Matteo Ferro, Savio Domenico Pandolfo, Felice Crocetto, Octavian Sabin Tataru, Riccardo Autorino, Michele Battaglia, Pasquale Ditonno, Giuseppe Lucarelli

**Affiliations:** 1 Urology, Andrology and Kidney Transplantation Unit, Department of Precision and Regenerative Medicine and Ionian Area, University of Bari “Aldo Moro”, 70124 Bari, Italy; 2 Division of Urology, European Institute of Oncology, IRCCS, 71013 Milan, Italy; 3 Department of Neurosciences and Reproductive Sciences and Odontostomatology, University of Naples “Federico II”, 80131 Naples, Italy; 4 Department of Simulation Applied in Medicine, George Emil Palade University of Medicine, Pharmacy, Sciences and Technology, 540139 Târgu Mureș, Romania; 5 Department of Urology, Rush University Medical Center, Chicago, IL 60612, USA

**Keywords:** renal cell carcinoma, tumor microenvironment, angiogenesis, metabolism, therapy

## Abstract

Globally, clear-cell renal cell carcinoma (ccRCC) represents the most prevalent type of kidney cancer. Surgery plays a key role in the treatment of this cancer, although one third of patients are diagnosed with metastatic ccRCC and about 25% of patients will develop a recurrence after nephrectomy with curative intent. Molecular-target-based agents, such as tyrosine kinase inhibitors (TKIs) and immune checkpoint inhibitors (ICIs), are recommended for advanced cancers. In addition to cancer cells, the tumor microenvironment (TME) includes non-malignant cell types embedded in an altered extracellular matrix (ECM). The evidence confirms that interactions among cancer cells and TME elements exist and are thought to play crucial roles in the development of cancer, making them promising therapeutic targets. In the TME, an unfavorable pH, waste product accumulation, and competition for nutrients between cancer and immune cells may be regarded as further possible mechanisms of immune escape. To enhance immunotherapies and reduce resistance, it is crucial first to understand how the immune cells work and interact with cancer and other cancer-associated cells in such a complex tumor microenvironment.

## 1. Introduction

Renal cell carcinoma (RCC) accounts for about 3–5% of all human cancers, and according to the 2023 American Cancer Society’s estimates, about 81,800 new cases will be diagnosed in the USA and 14,890 patients will die from this cancer [1]. Globally, clear-cell renal cell carcinoma (ccRCC) represents the most prevalent type of kidney cancer. Transcriptomic studies have supported the hypothesis that the proximal tubular epithelial cell (PTEC) is the cell of origin of the ccRCC [2,3,4]. Because changes in metabolic pathways contribute to its development, ccRCC is considered a cell metabolism disease [5,6,7,8]. In ccRCC, cancer cells develop a range of metabolic alterations that support their uncontrolled growth and proliferation. One such alteration is the activation of the hypoxia-inducible factors (HIFs) pathways, which increase glucose uptake and alter the cellular metabolism to produce energy in an oxygen-independent manner [9,10,11,12,13]. RCC cells also display an altered lipid metabolism, which is characterized by an increased uptake of fatty acids and significant accumulations of polyunsaturated fatty acids [14,15].

Surgery plays a key role in the treatment of this tumor, although one third of patients are diagnosed with metastatic ccRCC and about 25% of patients will develop a recurrence after nephrectomy with curative intent [16,17,18,19]. In this scenario, it is urgent to identify novel biomarkers not only for diagnostic purposes but also for prognostic and predictive factors of response to therapy [20,21,22,23,24]. Furthermore, the recent introduction of imaging techniques based on artificial intelligence algorithms will be of considerable support for the risk stratification, treatment selection, follow-up strategy, and prognosis of this tumor [25,26,27].

Molecular-target-based agents, such as tyrosine kinase inhibitors (TKIs) and immune checkpoint inhibitors (ICIs), are recommended for advanced cancers. However, a heterogeneous tumor microenvironment (TME) may promote resistance to these systemic therapies [28,29]. We describe the key characteristics of ccRCC TME in this review to offer potential directions for future therapeutic approaches.

## 2. Tumor Microenvironment

In addition to cancer cells, the tumor microenvironment (TME) includes non-malignant cell types embedded in an altered extracellular matrix (ECM). The composition of the TME varies between tumor types, but common features include a variety of cells (fibroblasts, adipocytes, neurons, endothelial cells, immune cells, and stem cells) and secreted molecules (cytokines, chemokines, growth factors, etc.). Deeper cataloging and comprehension of this context have been allowed because of novel techniques such as single-cell transcriptomic sequencing. Over the past years, different studies have linked patients’ prognoses and therapy responses to the RCC TME composition. The evidence confirms that interactions among the cancer cells and TME elements exist and are thought to play crucial roles in the development of cancer, making them promising therapeutic targets [30]. Hence, by producing growth factors or cytokines and by altering the TME (hypoxia and necrosis), RCC cells may promote non-tumor cells’ attraction and activation [31].

### 2.1. Cancer-Associated Fibroblasts (CAFs)

**Figure 1:**
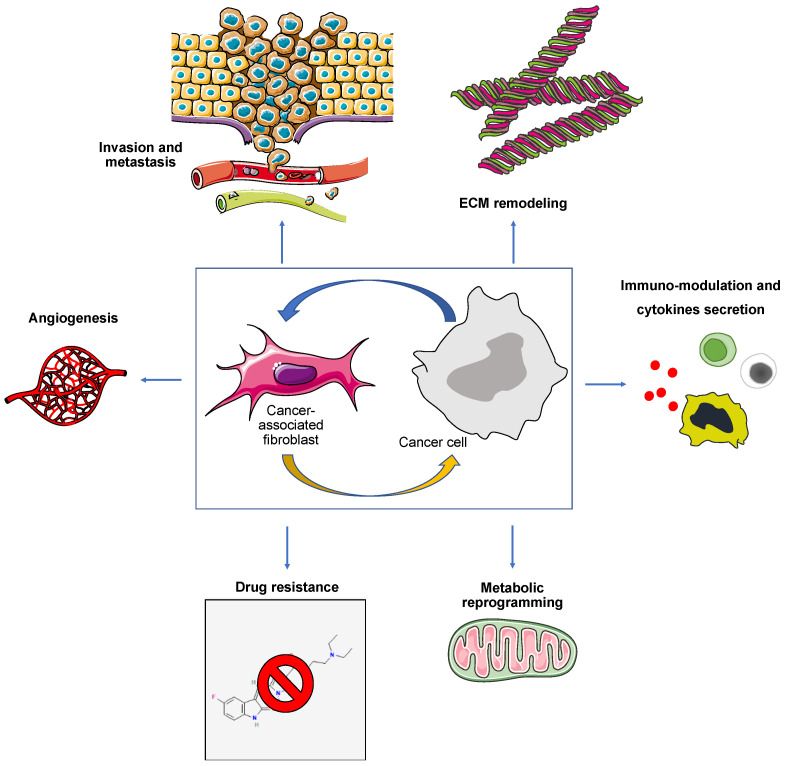
Summary of cancer-associated fibroblast biological functions.

Different theories have been proposed for the origin of cancer-associated fibroblasts (CAFs). They are described to be less abundant in RCC than in other solid cancers. Transforming growth factor-β (TGF-β), platelet-derived growth factor (PDGF), IL-1, IL-6, and TNF-α seem to be involved in their recruitment. Resident fibroblasts may give rise to CAFs. Large amounts of TGF-β are then released by the CAFs, thus initiating an autocrine signaling loop. In vitro and in vivo studies have already suggested that CAFs may also arise from adipose-derived stem cells (ASCs), endothelial cells, cancer epithelial cells (as a result of epithelial-to-mesenchymal transition, or EMT), and bone marrow mesenchymal stem cells (MSCs) [32]. The aberrant expression of smooth muscle actin (α-SMA), fibroblast-specific protein-1 (FSP1 or S100A4), vimentin, desmin, platelet-derived growth factor receptor (PDGFR)-α and -β, and fibroblast-activation protein-α (FAP) characterize these cells. Vascular endothelial growth factor (VEGF), PDGF, TGF-β, epidermal growth factor (EGF), fibroblast growth factor (FGF), hepatocyte growth factor (HGF), stromal-derived factor-1α, and osteopontin are known to be secreted by CAFs in the TME. The extracellular matrix (ECM) is a non-cellular structural component of the TME. Laminin, fibronectin, collagen type IV, nest protein, and proteoglycan are just a few of the elements that make up this structure in RCC. By serving as a substrate for cell adhesion and motility and as a reservoir for the sequestration of released molecules, the ECM promotes intercellular communication in the TME. Preclinical investigations have demonstrated that CAFs directly inhibit T-cell recruitment or activation by secreting CXCL12 and TGF-β or by creating a physical barrier through the deposition of ECM. Therefore, they are linked to T-cell dysfunction and exclusion [33,34]. CAFs may secrete galectin-1 (Gal1), which was noted to provoke CD8 T cells’ apoptosis. In gastric cancer, Gal1 has also been reported to promote EMT [35]. Moreover, antitumor immunity may be interfered with indirectly because immunosuppressive myeloid cells and T regs may be recruited by secreted mediators (i.e., IL-6, IL-1 β, etc.). CAFs have a role in tumor cell metabolic reprogramming, EMT induction, survival pathways, cancer invasion and metastasization, angiogenesis, drug resistance, immunomodulation, and cytokine secretion (Figure 1).

An increased expression of the genes involved in the EMT pathway is described in locally invasive ccRCC because of CAFs causing an ECM remodeling within the lesions and then facilitating the tumor spreading. Previous studies have demonstrated the CAFs’ heterogeneity within the tumor bulk in different cancer types: i.e., myofibroblasts (myCAFs), inflammatory CAFS (iCAFs), and antigen-presenting CAFs (ap-CAFs) [36,37]. The epithelial-to-mesenchymal transition is referred to as a reversible process by which fully differentiated cells lose their epithelial features and develop a migratory mesenchymal phenotype. Upregulation of ZEB1, ZEB2, Snail, Twist, and Slug leads to E-cadherin loss, which is considered a crucial step during EMT. The c-MET/MAPK, Wnt/β-catenin, PI3K/AKT, and JAK/STAT pathways have been shown to drive mesenchymal traits in RCC. Some of these signaling pathways depend on growth factor receptors. So, as in breast and pancreatic cancer, MUC1 is involved in EMT because it suppresses E-cadherin expression [38,39]. Moreover, EMT initiates the sarcomatoid conversion of ccRCC, which is characterized by E- to N-cadherin switching, membrane dissociation of β-catenin, and enhanced expression of Snail and Sparc [40].

### 2.2. Tumor Vascular Cells

Lower levels of adhesion molecules are expressed by the tumor endothelial cells (TECs), thus impairing the barrier function as does the reduced interaction between the TECs and pericytes. Pericytes also interact with other stromal cells and cancer cells, modulating the TME [41]. Tumor blood vessels are notably characterized by irregular branching, tortuous course, arteriovenous shunting, and an altered surface area to volume ratio [42]. Leaky and disorganized tumor vessels also affect cell oxygenation and immune cell dysfunction, and reduce drug penetration. Upon binding to its receptor (VEGF 1-2-3), VEGF activates downstream messengers, which lead to the expression of genes responsible for the proliferation, survival, migration, and permeability of the vascular endothelial cells. VEGFR is coupled to an intracellular tyrosine or serine/threonine kinase. mTOR is an essential part of the PI3K/AKT signaling system, which controls several biological processes such as protein synthesis, angiogenesis, and autophagy. Deregulation of mTOR signaling is related to the development of cancer [43]. Tumor-associated myeloid cells (i.e., neutrophils and macrophages) may enhance angiogenesis via pro-angiogenic mediators, including VEGF, FGF2, PIGF, and BV8. The RCC cells recruit mast cells and cancer endothelial cells through modulating the PI3K/AKT/GSHβ/AM signaling [44]. Cytogenetic abnormalities (aneuploidy) have been described in TECs in RCC. In association with high glycolytic activity, this finding reflects a hyperactivated phenotype, although TECs have always been thought not to be able to proliferate [45]. In addition, the androgen receptor (AR) may promote angiogenesis by recruiting endothelial cells in RCC via the AKT/NF-kB/CXCL5 axis [46]. Lymphatic endothelial cells (LECs) cover the walls of lymphatic vessels, representing a dissemination route for cancer cells. TECs and LECs may express immune checkpoint molecules such as PD-L1 (programmed-death-ligand-1), IDO1, and TIM3. At the same time, LECs may present tumor antigens in the absence of co-stimulatory signals. For these reasons, LECs and TECs have been recognized as possible regulators of antitumor immunity and immunotherapy response [47,48].

### 2.3. Tumor-Associated Adipocytes

The surrounding adipose microenvironment may regulate the activity of tumor and non-tumor renal epithelial cells. Adipocytes have been reported to release free fatty acids, hormones, cytokines, adipokines, and growth factors, which may impact cancer progression [49,50]. Therefore, they may promote a pro-tumorigenic low-grade chronic inflammation [51]. Adiponectin gene polymorphism rs182052 is associated with ccRCC risk, and leptin receptor gene polymorphism rs1137101 may also be a possible risk factor for RCC [52,53]. Robust glycogen and lipid accumulation is observed in ccRCC. Lipid droplets store cholesterol esters and triglycerides within the cancer cells. Recently, Ferrando et al. compared human adipose explants from normal (hRAN) and kidney cancer (hRAT) tissue. A higher expression of leptin and its receptor (ObR) and smaller adipocytes were noted in hRAT than in hRAN. These findings may relate to increased lipolysis and therefore increased energy availability in hRAT. Because leptin is known to induce a fibroblastoid morphology in breast cancer, it is speculated that it may contribute to the upregulation of EMT markers in RCC [54,55].

### 2.4. Tumor Immune Microenvironment

NK cells, effector T cells, and mature dendritic cells are tumor-associated immune cells that may be involved in the anticancer immune response, whereas regulatory T cells and myeloid-derived suppressor cells (MDSCs) have the opposite impact.

#### 2.4.1. T Cells

Antigen-presenting cells (APCs) such as dendritic cells and their major histocompatibility complex (MHC) are necessary for T cells’ activation. The T-cell receptors (TCR) recognize the antigen peptides in MHC; CD8 T cells bind class I MHC, whereas CD4 T cells bind class II MHC. The T-cell coreceptors CD4 and CD8 bind the non-polymorphic domains of MHC. However, co-stimulation is required to fully activate effector T cells: CD28 binds B7-1 (CD80) or B7-2 (CD86) on APCs or B cells. CD8 T cells destroy their targets via granzyme, perforin-mediated apoptosis, or via the FAS-FASL axis [56]. CD4 helper T cells affect a variety of other immune cells. According to their phenotype, CD4 T cells may exert dual effects. For instance, the Th1 subtype enhances CD8 T cells and B cells, and it may directly kill cancer cells via IFN-γ or TNF-α. On the opposite side, the Th2 subtype releases anti-inflammatory mediators, thus limiting antitumor responses. Immune checkpoint molecules represent pivotal elements involved in the cancer immune escape. PD-L1 on the renal epithelial cancer cells binds T cells’ inhibitory receptor PD-1 (programmed death-1). Upon binding, the activated T cells either die or lose their function. Another PD-1 ligand, PD-L2 (also known as B7-DC), is known to be expressed by tumor-infiltrating dendritic cells. Activated T cells may express CTLA-4 (cytotoxic T lymphocyte antigen-4), which provides negative feedback signals for T-cell activation at the lymph node level. Other immune checkpoint molecules (B7-H3, B7-H4, VISTA, PD-1H, TIM-3, LAG-3, TIGIT, etc.) have been identified, and clinical studies have evaluated their clinical relevance [57,58,59]. A continuous transition to terminally exhausted clonotypes has been found using transcriptome analysis of CD8 T cells in ccRCC. Indeed, naïve, cytotoxic, exhausted, progenitor, and terminally exhausted T cells have been isolated. In advanced and metastatic ccRCC microenvironments, higher exhausted T cells with low TCR diversity were inferred than in normal kidney samples or the peripheral blood [60,61]. Giraldo et al. investigated the associations among the infiltration of CD8 T cells and mature dendritic cells (DCs), the expression of immune checkpoint molecules, and the patients’ prognosis. A poor prognosis characterized the first group, with a strong expression of immune checkpoint molecules and low mature DCs. In turn, mature DCs and a lower expression of immune checkpoint molecules were associated with a better prognosis [62]. The same group then identified three immune profiles of ccRCC: immune-regulated (CD8 PD-1+TIM-3+LAG-3+ TILs and T regs), immune-activated (CD8 PD-1+ TIM-3+ TILs), and immune-silent (enriched with TILs similar to those in the adjacent non-malignant tissue). Remarkably, the immune-regulated tumors had a significant risk of disease progression and had aggressive histologic features [63]. FoxP3 regulatory T cells (T regs) are a subpopulation of CD4 T cells with immunosuppressive properties in the TME. These cells work in the healthy host to promote immunological homeostasis and self-tolerance. In turn, these cells contribute to suppressing effective antitumor immunity via different mechanisms, including the expression of cytokines (IL-2, IL-10, TGF-β, adenosine), direct cytotoxicity (perforin and granzyme), promotion of tolerogenic dendritic cells with a reduced capacity to activate effector T cells, and augmentation of T-reg production [64,65,66]. Immune checkpoint molecules (PD-1, CTLA-4, Tim-3, LAG3, TIGIT, etc.) may be expressed by FoxP3 T regs to further limit anticancer responses. As a consequence, the CD8 T-cell population increases and tumor growth slows upon reducing the T-reg population in the TME [67].

#### 2.4.2. Tumor-Associated Myeloid Cells

Tumor-associated macrophages (TAMs) may arise from different sources, such as tissue-resident macrophages or bone-marrow-derived infiltrating ones. Macrophages and neutrophils are phagocytic effectors of anticancer innate immunity. The simple binary states of classical versus alternative activation were first used to categorize myeloid cells. Macrophage polarization not only depends on intrinsic signaling (ERK, NF-kB, and STAT1 vs. STAT3 and STAT6 pathways) but it is also regulated by immune, stromal, and cancer cells in the TME in a context-dependent manner [68]. M1 polarization depends on Th1 cytokines (IFN-γ); M1 macrophages release pro-inflammatory cytokines such as IL-6, IL-12, IL-23, and TNF-α and toxic compounds (i.e., reactive oxygen species-ROS). Th2 cytokines (IL-4, IL-10, and IL-13) promote M2 macrophages, which are known to reduce inflammation and promote angiogenesis, wound healing, and tissue remodeling. M2-TAMs are typically characterized by an impaired antigen presentation but an increased expression of angiogenic factors (VEGF), tissue remodeling metalloproteases (MMPs), cathepsins, TGF-β, IL-10, prostaglandin E2, and other molecules that may limit lymphocyte and macrophage proliferation and function. Therefore, after a tumor has developed, M2-TAMs may dampen immune surveillance and alter the ECM to accelerate tumor growth [69]. Similar to TANs, immune checkpoint molecules may be expressed by TAMs, which may also use T regs to further suppress antitumor immunity [70]. Upon binding to signal regulatory protein-α (SIRP-α, an inhibitory receptor on phagocytes), CD47 on tumor cells may block phagocytosis. Worse prognoses and more aggressive phenotypes of ccRCC have recently been linked to CD47 expression [71,72]. Multiple TAM phenotypes have been described, and different TAM subsets may coexist within tumors [73]. Different transcriptomic patterns are displayed by the macrophage subgroups among the different tumor types. This supports the concept that tumor-associated myeloid cells are imprinted according to the organ and cancer type. In ccRCC specimens, a continuum from M1-like to M2-like states was shown by the TAM populations. It is well established that TAMs are involved in different processes of tumorigenesis, ranging from initiation to angiogenesis, metastasis, and immune escape. CCL2 may recruit macrophages to hypoxic regions of the tumors, where increased HIF1α/HIF2α induces the transcription factor of several angiogenesis-related genes. Here, the TAMs may release growth factors, cytokines, MMPs, and other molecules that promote blood vessel formation and stabilization [74]. Hence, a high CD68+ TAM density has been associated with a high microvessel density [75]. Previous studies have reported that a higher number of TAMs in the TME is associated with poorer prognoses and earlier relapses in RCC patients [76,77]. Chittezhath et al. showed that IL-1-IL-1R signaling is crucial for controlling the tumor-promoting phenotypes of the monocytes and macrophages in RCC. Therefore, further investigations are required to uncover the potential therapeutic role of anti-IL-1 for selected human cancers [78].

Tumor-associated neutrophils (TANs) are also categorized as N1 (antitumor) or N2 (pro-tumor), depending on their effects. Direct or antibody-dependent cytotoxicity, along with the stimulation of several innate and adaptive immune cells (NK cells, B and T cells, and DCs), are the main mechanisms through which N1 action against tumors is exhibited [79]. In a murine model of RCC, N1-TANs were shown to build an antimetastatic barrier, thus limiting cancer spreading to the lungs [80]. In turn, N2-TANs may promote, directly or indirectly, tumor growth, angiogenesis, and metastasis. Several cytokines have been shown to guide neutrophils’ recruitment in mice and in human solid cancers. IFN-γ has been noted to stimulate N1 polarization, whereas TGF-β promotes N2-TANs [81]. A high neutrophil to lymphocyte ratio (NLR) in both the peripheral blood and the TANs is linked to a poor prognosis in RCC patients [82]. Intriguingly, Song et al. found that N2-TANs may promote the progression of RCC via the androgen receptor/c-Myc pathway [83].

Myeloid-derived suppressor cells (MDSCs) represent a heterogeneous group of myeloid cells [84]. According to their origin, from granulocytic or monocytic myeloid cell lineages, granulocytic/polymorphonuclear MDSCs (PMN-MDSCs) and monocytic MDSCs (M-MDSCs) are the two main categories of MDSCs in humans and mice. Pro-inflammatory mediators (prostaglandin E2, IL-6, VEGF, and complement fragment C5a) and growth factors (GM-CSF, M-CSF) are demanded for their recruitment and activation from the bone marrow at tumor sites. While M-MDSCs use nitric oxide (NO) and immunosuppressive cytokines (IL-10 and TGF-β), as well as the expression of immune checkpoint molecules such as PD-L1, PMN-MDSCs preferentially use reactive oxygen species (ROS), peroxynitrite, and prostaglandin E2 (PGE2) to mediate immune suppression. Additionally, MDSCs may enhance cancer immune escape via the deprivation of essential amino acids such as cysteine, arginine (Arg), and tryptophan (TRP) because they may express arginase-1 and indolamine 2,3-dioxygenase 1 (IDO1) [85,86,87]. MDSCs may also play a crucial role in the formation of the premetastatic niche. The chemokine receptors CXCR2 and CXCR4 are primarily responsible for attracting neutrophils or PMN-MDSCs to the premetastatic niches. By inhibiting immune cells, inducing ECM remodeling, and angiogenesis, MDSCs may facilitate the engraftment of tumor cells in the premetastatic niche [88].

## 3. Metabolic Reprogramming and Immune Escape in the TME

Large amounts of energy are required for tumor cells’ growth, proliferation, and metastasis. Oncogenic signals affect the metabolic pathways in cancer cells: increased glycolysis, glutaminolysis, and lipolysis support bioenergetic demands. Increased utilization of glutamine is often found in ccRCC to generate citrate and lipids. Indeed, glutamine, cysteine, or glutamate deprivation may be beneficial for the treatment of Von Hippel–Lindau (VHL)-deficient RCC [89]. Despite oxygen availability, they typically show aerobic glycolysis (the Warburg effect), which is responsible for TME acidification because of lactate accumulation. Lactate may suppress the activation of effector T cells and limit the differentiation of monocytes and DCs, whereas it promotes T regs and the M2-like phenotype of TAMs [90,91]. An unfavorable pH, waste product accumulation, and competition for nutrients between cancer and immune cells may be regarded as further possible mechanisms of immune escape [92,93]. Nutrients’ availability in the TME also depends both on systemic (the patient’s diet and nutritional state) and local factors (the tumor type and its location within the primary tissue) [94,95]. pVHL loss and HIF1α stabilization promote the expression of glycolytic transporters and enzymes such as GLUT1, hexokinase 1 (HK1) and 2 (HK2), pyruvate kinase muscle isozyme 2 (PKM2), pyruvate dehydrogenase kinase 1 (PDHK1), and lactate dehydrogenase A (LDHA). A metabolomic analysis of ccRCC revealed different distributions of intermediates between the upper and lower parts of the glycolytic flux. A significant reduction in metabolites of the lower chain was noted because metabolites are rerouted toward the pentose phosphate pathway (PPP). NADH dehydrogenase (ubiquinone) 1 alpha subcomplex 4-like 2 (NDUFA4L2) inhibits Complex I of the electron transport chain (ETC). It is significantly overexpressed in ccRCC, as it is under the control of HIF1α. Mitochondrial dysfunction is thought to be a hallmark of cancer cells. Abnormal mitochondrial numbers and morphology; dysfunctional ETC; mitochondrial DNA (mtDNA) mutations; and oxidative damage to lipids, proteins, and nucleic acids are some of their distinguishing features. Several studies have noted the crosstalk between HIF1α accumulation and mitochondrial dysfunction in different cancer types. HIF1α limits triglyceride lipase-mediated lipolysis by HIG2, thus reducing fatty acid oxidation. Additionally, HIF1α delays ETC via NDUFA4L2, COX4-2, Complex I, and Complex IV [96]. Decreased cell viability, increased cisplatin susceptibility, inhibition of autophagic machinery, increased mitochondrial mass, and ROS accumulation were found after silencing or knocking down NDUFA4L2 [12]. The process of autophagy, which involves the fusion of vesicles (autophagosomes) with lysosomes with hydrolytic enzymes, allows cells to degrade and recycle proteins and organelles. Autophagy may be promoted by starvation and oxidative stress conditions to sustain metabolic demands; therefore, it may sustain cancer cells’ growth. When selective for mitochondria, it is referred to as mitophagy. It is well established that such a complex process involves different proteins (such as the autophagy-related Atg proteins) and that it modulates interactions between cancer cells and non-cancer cells in the TME. Two different pathways of mitophagy have been unveiled so far: PINK1/Parkin (depending on membrane depolarization) and BNIP3/NIX/FUNDC1 (depending on hypoxia). Of note, HIF1α upregulates BNIP and NIX expression [97,98,99].

In the TME, tumor cells may compete with CD8 T cells for different amino acids such as arginine, tryptophan, serine, cysteine, and alanine. Increased uptake of arginine by cancer cells, and its consumption by TAMs (via arginase-1), reduce its availability in the TME. Reduced mTORC activity in the T cells results in reduced T cells’ effector functions and increased memory-like T cells [100,101]. Extracellular serine has been demonstrated to be essential for the growth and effector capabilities of T cells, which are compromised when serine levels are low in the TME [102].

Three metabolic pathways consume tryptophan (TRP): protein synthesis, serotonin, and kynurenine (KYN) production [103]. Two enzymes are known to transform TRP to KYN: tryptophan 2,3-dioxygenase (TDO) and indoleamine 2,3-dioxygenase (IDO1). The expression of IDO1 may also be induced by TNF-α and IFN-γ. Riesenberg et al. explored the role of IDO1 expression in TECs in ccRCC. They identified an increased microvascular density in tumors with higher IDO+-TECs [104]. Chen et al. reported the upregulation of TDO in CAFs in renal cancer [105]. In addition to TRP depletion in the TME, the tumorigenic function of KYN appears to be mediated by its interaction with aryl hydrocarbon receptors (AhR) on immune and cancer cells. The KYN/AhR axis may facilitate cancer cells’ survival, migration, and chemoresistance. Immunosuppressive T reg cells are differentiated because of the activation of AhR in CD4 T cells [106]. Additionally, PD-1 expression on CD8 T cells is induced by KYN [107]. Recently, the authors have investigated the role of MUC1 in the TME of ccRCC. In MUC1^H^ tumors, M2-like TAMs (CD68+CD163+) are able to produce KYN. Moreover, MUC1^H^ samples have shown increased deposition of C1q, which colocalized with pentraxin-3 (PTX3), in association with higher expression of proangiogenic receptors (C3aR and C5aR). PTX3 is known to activate the classical cascade of the complement system [108,109]. Nonetheless, the increased expression of CD59 limited C5b-9 assembly in the TME of MUC1^H^ ccRCC. Finally, this study demonstrated a lower expression of PD-L1 in MUC1^H^ samples [110].

## 4. Clinical Role of the TME and Therapeutic Implications

The balance of pro- and anti-angiogenic signals that regulates angiogenesis is referred to as the “angiogenic switch.” Pro-angiogenic signals (VEGF-A, the FGF receptor family, and MMPs) are counteracted by anti-angiogenic factors such as thrombospondin 1 and 2, angiopoietin, endostatin, osteopontin, angiostatin, and cellular communication network factor 3 (CCN3) [111]. Anti-angiogenic signals are overcome because angiogenesis is a defining characteristic of malignancies. Somatic mutations of the Von Hippel–Lindau (VHL) gene are observed in approximately 92% of patients diagnosed with ccRCC. VHL loss leads to a constitutive activation of hypoxia-induced response elements (HRE), genes involved in metabolism (GLUT1, PDK1, and EPO), proliferation, cell survival, and angiogenesis (VEGF and PDGF). Because of its crucial role, anti-angiogenic therapies have been developed for the treatment of patients with advanced clear-cell and non-clear-cell RCCs [112].

### 4.1. Angiogenesis Inhibitors

Bevacizumab is a recombinant humanized monoclonal antibody that prevents circulating VEGF from binding to its receptor on the endothelial cell surface [113,114]. In 2003, it showed superiority as a single agent in metastatic ccRCC compared with placebo. Then, its use in combination with IFNα was approved in a metastatic setting. This combination is recommended by the European Society of Medical Oncology (ESMO) guidelines in metastatic ccRCC patients with a good or intermediate prognosis [115]. Subsequently, different oral anti-angiogenic tyrosine kinase inhibitors (TKI) have been introduced for the treatment of advanced RCC. TKIs have different targets and several sites of action. For instance, sunitinib blocks VEGFR and PDGFR tyrosine kinases, as well as FMS-like tyrosine kinase 3 (Flt-3), colony-stimulating factor 1 receptor (CSFR1), and neurotrophic factor. These tyrosine kinases affect not only angiogenesis, but also tumor growth and metastatic progression. In a phase III study, sunitinib overcame IFNα-2a in terms of progression-free survival (PFS), objective response rate (ORR), and quality of life (QoL) [116,117]. Pazopanib targets VEGFR, FGFR, PDGFR, and c-Kit, limiting angiogenesis and tumor growth [118]. In a phase III clinical trial, it demonstrated PFS and ORR benefits over placebo, so that pazopanib has been approved as a first-line therapy for metastatic ccRCC [119]. Sorafenib is active against VEGFR, PDGFR, c-Kit, Flt-3, and RET-receptor kinases, thus decreasing angiogenesis and cell replication. However, when tested against placebo in a phase III study, it demonstrated benefits in PFS but not overall survival (OS). Therefore, it has been approved for the treatment of advanced ccRCC patients who failed prior INFα or IL-2 therapy [120]. Axitinib is a second-generation TKI against VEGFR that has been approved for metastatic ccRCC when prior sunitinib or cytokine treatment has failed [121,122]. In a phase IIII study, cabozantinib provided a better PFS and ORR than everolimus in the CABOSUN trial when compared with sunitinib. Several cabozantinib targets are known so far (VEGFR2, MET, ROS1, TYRO3, Flt-3, c-Kit, RET, AXL, etc.), which are involved in cancer progression at different levels [123]. It has been approved as a first-line therapy for metastatic ccRCC patients with poor or intermediate prognosis and as second-line treatment in case of prior failed VEGF-targeted therapies [124].

### 4.2. Mammalian Target of Rapamycin (mTOR) Inhibitors

As mentioned above, mTOR plays a crucial role in the PI3K/AKT axis, which controls angiogenesis, cell proliferation, and metabolism. Additionally, HIF expression is also promoted by mTOR. Therefore, mTOR inhibition has been introduced as a target in RCC [125]. Due to their superior efficacy and tolerability, various targeted and ICI treatments have replaced it in clinical practice. Temsirolimus was approved by the EMA as a single drug for the first-line treatment of adult patients with at least three out of six negative prognostic factors according to the MSKCC classification [126]. On the basis of the RECORD I research, everolimus was approved for use in the treatment of ccRCC that had progressed after receiving first-line therapy [127].

### 4.3. Cytokine Therapy and Immune Checkpoint Inhibitors (ICIs)

Although chemoresistant, RCCs have long been thought to be highly immunogenic. Spontaneous remissions of metastatic ccRCC patients after surgery were observed in the 1960s. Immunotherapy drugs enhance the host’s antitumor immune responses rather than directly destroying their targets. IL-2 and IFN-α were the initial immunotherapy regimens used to treat metastatic RCC. A better understanding of immune escape mechanisms led to the development of antibodies against immune checkpoint molecules, which are currently used for advanced RCCs. Ipilimumab (anti-CTLA-4) was the first ICI to be introduced for the treatment of metastatic ccRCCs [128]. When compared with everolimus, nivolumab (anti-PD-1) had superior tolerability and an improved OS and ORR [129]. However, the combination of ipilimumab and nivolumab has shown promising efficacy and greater response rates than either agent alone. Over time, clinical trials have evaluated the role of pembrolizumab (anti-PD-1), atezolizumab, avelumab, and spartalizumab (PD-L1 inhibitors) as therapeutic agents for ccRCC. The efficacy of PD-L1 as a prognostic marker for mccRCC is still debated, even though elevated PD-L1 expression appears to be predictive of responsiveness to checkpoint inhibitors. PD-L1 positive patients seem to respond better to anti-PD-1/PD-L1 agents than PD-L1 negative patients, although both groups benefit from ICI when compared with the sunitinib group [130]. Preclinical research has shown that angiogenic inhibition can increase T-cell infiltration into tumors, increasing the efficacy of ICI [131]. This rationale paved the way to phase III studies exploring this combination approach (Table 1) [132,133,134,135,136]. Recent European Association of Urology (EAU) guidelines consider ICI and TKI combination therapy as a first-line treatment for metastatic RCC. The International Metastatic RCC Database Consortium (IMDC) scale is applied to stratify patients according to their predicted prognosis [137].

Finally, belzutifan, the first HIF inhibitor, has been approved for use in advanced ccRCC with VHL disease, and further studies are evaluating its clinical efficacy in association with ICI and other targeted therapies [138].

## 5. Conclusions

A significant number of patients diagnosed with advanced-stage RCC remain unresponsive or even develop resistance to the currently available systemic therapies. To enhance immunotherapies and reduce resistance, it is crucial to first understand how the immune cells work and interact with the cancer and other cancer-associated cells in such a complex tumor microenvironment. Exploring the different metabolic pathways in the TME may give novel approaches to reduce immune suppression and to limit metastasis. Developing novel predictive biomarkers, adopting the optimal therapeutic regimens, or combining them in accordance with risk models might be useful to improve survival outcomes, therapeutic safety, and quality of life of RCC patients.

## Figures and Tables

**Table 1:** Phase III clinical trials evaluating therapeutic combinations of immune checkpoint inhibitors and anti-angiogenic agents for advanced-stage ccRCC. PFS: progression-free survival; OS: overall survival.

Trial	Drugs	Primary Endpoint	
NCT02420821	Atezolizumab + Bevacizumab	PFS	[132]
NCT02853331	Pembrolizumab + Axitinib	PFS, OS	[133]
NCT02684006	Avelumab + Axitinib	PFS, OS	[134]
NCT02811861	Pembrolizumab + Lenvatinib	PFS	[135]
NCT03141177	Nivolumab + Cabozantinib	PFS	[136]

## Data Availability

No new data were created.
